# TRIM3 attenuates cytokine storm caused by *Dabie bandavirus via* promoting Toll-like receptor 3 degradation

**DOI:** 10.3389/fmicb.2023.1209870

**Published:** 2023-07-14

**Authors:** Ke Jin, Yan Dai, Ke Ouyang, Huaying Huang, Zhengyi Jiang, Zhan Yang, Tingting Zhou, Hong Lin, Chunhui Wang, Chunyan Wang, Xuewei Sun, Dafeng Lu, Xiaoguang Liu, Nannan Hu, Chuanlong Zhu, Jin Zhu, Jun Li

**Affiliations:** ^1^Department of Infectious Disease, The First Affiliated Hospital of Nanjing Medical University, Nanjing, China; ^2^School of Integrated Chinese and Western Medicine, Nanjing University of Chinese Medicine, Nanjing, China; ^3^Epidemiological Department, Huadong Medical Institute of Biotechniques, Nanjing, China; ^4^Department of Transfusion Research, Jiangsu Province Blood Center, Nanjing, China; ^5^School of Basic Medicine, Binzhou Medical University, Yantai, China; ^6^School of Public Health, Nanjing Medical University, Nanjing, China; ^7^Department of Respiratory and Critical Care Medicine, Jinling Hospital, The First School of Clinical Medicine, Southern Medical University, Nanjing, China

**Keywords:** severe fever with thrombocytopenia syndrome, *Dabie bandavirus*, TRIM3, Toll-like receptor 3, cytokine storm

## Abstract

**Background:**

Severe fever with thrombocytopenia syndrome (SFTS) is an emerging infectious disease that was caused by the *Dabie bandavirus* (DBV), and it has become a global public health threat. Cytokine storm is considered to be an important pathogenesis of critical SFTS. Tripartite motif-containing 3 (TRIM3), as a member of the TRIM protein family, may contribute to the regulation of the immune and inflammatory responses after viral infection. However, whether TRIM3 plays a major role in the pathogenesis of SFTS has not yet been investigated.

**Methods:**

TRIM3 mRNA levels were detected in PBMCs between 29 SFTS patients and 29 healthy controls by qRT-PCR. We established the pathogenic IFNAR^−/−^ SFTS mouse model successfully by inoculating subcutaneously with DBV and testing the expression levels of TRIM3 mRNA and protein by qRT-PCR and immunofluorescence in the livers, spleens, lungs, and kidneys. TRIM3^OE^ THP-1 cells and peritoneal macrophages extracted from TRIM3^−/−^ mice were infected with DBV. The effect of TRIM3 on cytokines was detected by qRT-PCR and ELISA. Then we examined Toll-like receptor 3 (TLR3) and protein phosphorylation in the MAPK pathway after DBV infection using Western blot. Flow cytometry was used to verify TLR3 expression on peripheral blood monocytes in SFTS patients. We further explored the interaction between TRIM3 and TLR3 using CO-IP and Western blot.

**Results:**

Compared to healthy controls, TRIM3 mRNA expression in PBMCs is decreased in SFTS patients, especially in severe cases. TRIM3 mRNA and protein were synchronously reduced in the livers, spleens, lungs, and kidney tissues of the IFNAR^−/−^ SFTS mice model. In the DBV-infected cell model, TRIM3 overexpression can inhibit the DBV-induced release of IL-1β, IL-6, and TNF-α, the expression of TLR3, and protein phosphorylation in the MAPK pathway, which plays an anti-inflammatory role, while TRIM3 deficiency exacerbates the pro-inflammatory effects. We further found that TRIM3 can promote TLR3 degradation through K48-linked ubiquitination.

**Conclusion:**

TRIM3 can inhibit the production of cytokines by regulating the degradation of TLR3 through K48-linked ubiquitination, which can be a therapeutic target for improving the prognosis of SFTS.

## 1. Introduction

Severe fever with thrombocytopenia syndrome (SFTS) is an emerging infectious disease that is caused by *Dabie bandavirus* (DBV) (Yu et al., 2011; Li Y. H. et al., 2023). The population is generally susceptible. The average case fatality rate was 5.3–7.8% in China (Li, 2015; Zhan et al., 2017) and 32.6–50.0% in Japan and South Korea (Takahashi et al., 2014; Choi et al., 2016). Because of its epidemic and high mortality rate, this disease has become a global public health threat. Favipiravir, a GTP-competitive inhibitor of the viral polymerase, has been proven to reduce the case fatality rates of SFTS patients, which is only limited to the low viral load group (Li et al., 2021; Suemori et al., 2021; Yuan et al., 2021). Therefore, it is urgent to explore the pathogenesis and find therapeutic targets to decrease mortality.

Our previous studies have revealed that cytokine storms, which are caused by an exaggerated immune response, are responsible for organs and tissue damage and drive the process of SFTS development (Li et al., 2014). Zhang et al. (2019) also confirmed that in the early stage of infection, DBV induces the immune response of monocytes by activating STAT1, stimulates macrophages to differentiate into M1 phenotypes, and leads to the production of pro-inflammatory cytokines (such as TNF-α, IL-1β, and IL-6). Furthermore, in SFTS patients, abnormal production of inflammatory mediators was most associated with the severity of the disease (Hu et al., 2018). Therefore, inhibition of cytokine storm is particularly vital for relieving the severity and controlling the progression of SFTS.

Tripartite motif proteins (TRIMs) belong to the E3 ubiquitin ligase family and contain up to 80 members in mammals (Vunjak and Versteeg, 2019). The TRIMs family consists of three relatively conservative domains motif (RBCC), namely, the RING domain, the B-box domain, and the coiled-coil domain, and participates in a variety of biological processes including differentiation, proliferation, transcription, and apoptosis in virus infection, immunoregulation, and cell stress responses (Khan et al., 2019; Vunjak and Versteeg, 2019). Tripartite motif-containing 3 (TRIM3), as a member of the TRIMs family, has been revealed to provide a post-translational mechanism for inflammation, apoptosis, and proliferation (Wang et al., 2019; Dong et al., 2020; Li W. et al., 2022; Zhuang et al., 2022); however, whether TRIM3 is involved in the immune and inflammatory response after viral infection, especially DBV infection, remains unclear.

In this study, we constructed DBV-infected models *in vivo* and *in vitro*, investigated whether TRIM3 has a pivotal function against the process of SFTS, and further observed the underlying molecular basis of TRIM3 acting on immune and inflammatory responses, which provided new strategies for the treatment of SFTS.

## 2. Methods and materials

### 2.1. Ethics statement

All virus-related experiments in our study were conducted in the biological safety level 2+ laboratory of the Eastern Theater Disease Control and Prevention Center. We collected blood samples from 29 SFTS patients ranging from 4 to 18 days after onset who were admitted to the Department of Infectious Disease, First Affiliated Hospital of Nanjing Medical University between January 2020 and November 2021. Among the 29 SFTS patients, 3 patients were in the fever stage, and 26 patients were in the multiple organ failure (MOF) phase based on the clinical manifestations and examinations. The viral load of SFTS patients was 3.03–9.12 lg copies/ml. According to previous research (Deng et al., 2013; Liu et al., 2017), we divided the SFTS patients into 7 non-severe cases and 22 severe cases, and 6 severe cases died. Detailed information on patients is present in Supplementary Table 1. In addition, blood samples were collected from 29 healthy individuals aged 18–55 years who donated blood at the Jiangsu Provincial Blood Center from October 2020 to October 2021. This study was performed with the approval of the Ethics Committee of the Institutional Review Board at the First Affiliated Hospital of Nanjing Medical University, and each participant signed an informed consent form. Animal experiments were approved by the Huadong Medical Institute of Biotechniques (HMIB) ethics committee and conducted as per HMIB's ethical regulations.

### 2.2. TRIM3 knockout (TRIM3^−/−^) mice and IFNAR knockout (IFNAR^−/−^) mice

Using the principle of homologous recombination, the TRIM3 gene was modified by targeting ES cells. The ES cell targeting vector was constructed using the Infusion method. After linearization, the vector was electrotransfected into C57BL/6J × 129S3 ES cells. Positive ES cell clones were amplified and injected into the blastocysts of C57BL/6J mice to obtain chimeric mice. Then these mice were maintained and bred in an environmentally controlled and specific pathogen-free (SPF) animal facility at the Eastern Theater Disease Control and Prevention Center. When these mice grew to 7–10 days, their tails were cut for gene identification of TRIM3^−/−^ homozygote.

IFNAR^−/−^ C57BL/6 mice were purchased and identified from Cyagen (Cyagen Biosciences Inc.). All mice were bred and maintained in an environmentally controlled and SPF animal facility at HMIB. Mice aged 6–8 weeks were randomly divided into three groups of five mice. On day 0, mice were inoculated subcutaneously with 100 μl 1.0 × 10^5^ TCID_50_/ml, and 1.0 × 10^6^ TCID_50_/ml virus solution, while the mock mice were inoculated with 100 μl of DMEM. Every mouse in each group was weighed daily, and the livers, spleens, lungs, and kidney tissues were obtained after death.

### 2.3. Viruses and cells

The Chinese DBV strain JS14 was provided by the Jiangsu Provincial Center of Disease Control and Prevention and was extensively proliferated using Vero cells. The viruses used for experiments were all confirmed to be alive through plaque testing, and they were properly packaged and stored in an ultra-low-temperature freezer.

THP-1 and Vero Cells were obtained from Huzhen Industrial Company (Shanghai, China) and Cell Signaling Technology (Beverly, MA, USA) separately. THP-1 cells were cultured in 1,640 medium with 10% FBS and 1% penicillin-streptomycin, while Vero cells were cultured in DMEM medium. Peripheral Blood Mononuclear Cells (PBMCs) were derived from the blood donor cell filters. They were separated by the FICOII separation solution. The separated PBMCs were resuspended in DMEM, laid flat in a 6-hole plate with 1.5 × 10^7^ cells per hole, and then put overnight in a 5% CO_2_ incubator. The DMEM medium was slowly sucked out and discarded the next day. Instead, they were cultured in a 1,640 medium containing 10% FBS and 1% penicillin-streptomycin. Peritoneal macrophages were derived from corresponding C57BL/6 mice (TRIM3^−/−^, IFNAR^−/−^).

### 2.4. Construction of lentivirus

The total length of the CDS region of the human *TRIM3* gene was found in GenBank. According to the CDS region of the *TRIM3* gene and the selected vector, enzyme digestion was performed on upstream EcoR I and downstream BamH I, and primers were designed. The primers and TRIM3 overexpression (TRIM3^OE^) Lentivirus were synthesized by Shanghai Genepharma Company. Lentivirus-mediated overexpression was constructed both in PBMCs and THP-1 cells successfully.

### 2.5. RNA extraction and reverse transcription quantitative PCR

TRIzol regent (Invitrogen, USA) was used for isolating total RNA, PrimeScript RT Master Mix (Takara, Japan) was performed for cDNA synthesis, and TB Green Premium Ex Taq (Takara, Japan) was used for real-time PCR on the ABI QuantStudio 5 sequence detection system. Primers for qPCR are shown in Supplementary Table 2. GAPDH was used as an endogenous control for relative mRNA expression.

### 2.6. Western blot

According to the manufacturer's instructions (BestBio, China), we extracted total proteins. All proteins were denatured at 100°C for 10 min, then separated by SDS-PAGE using 12% polyacrylamide gel, and finally transferred to a nitrocellulose membrane (Sangon Biotech, China). After blocking in Tris buffer saline (TBS) containing 4% fetal bovine serum albumin (BSA; Roche), the membrane was incubated with the first antibody and a corresponding horseradish peroxidase coupled secondary antibody, and then washed thoroughly using TBS-T (supplemented with 0.1% TBS of Tween 20, pH 7.4). Protein signals were detected using an enhanced chemiluminescence assay kit (ECL; Tanon) and visualized using a chemiluminescence imaging system (Tanon 5200, China). We put primary antibodies (TRIM3, ProteinTech; Toll-like receptor 3 (TLR3), K48, TRIF, TRAF6, TAB1, TAB2, phosphorylation p38, phosphorylation JNK1/2, phosphorylation IKKα/β, phosphorylation NF-κB, p38, JNK1/2, NF-κB, and GAPDH, Cell Signaling Technology) as target bands, and the anti-rabbit and/or anti-mouse IgG antibodies (Cell Signaling Technology, Beverly, MA, USA) as the secondary antibodies.

### 2.7. Coimmunoprecipitation and western blot

We carried out coimmunoprecipitation (CO-IP) and immunoblotting experiments as described below. For transient transfection and CO-IP experiments, THP-1 cells were lysed in 1 ml of NP-40 buffer (20 mM Tris HCl, pH 7.5150 MM NaCl, 1 mM EDTA, 1% NP-40, 10 μg/ml aprotinin, 10 μg/ml leucin peptide, and 1 mM phenylmethyl sulfonyl fluoride) and then centrifuged at 12,000 rpm for 10 min at 4°C. For each immunoprecipitation, the supernatant was kept at 4°C for 2 h with a 1:1 serum of 0.5 μg antibody or control IgG and 30 μl protein G Sepharose. Then, we washed the agarose beads three times with 1 ml of lysis buffer containing 500 mM NaCl and analyzed the sediment using standard immunoblotting with the indicated antibody.

### 2.8. ELISA

Following the instructions of the corresponding ELISA kit (R&D Systems, USA), we tested pro-inflammatory cytokines (IL-6, IL-1β, and TNF-α) in the cell supernatant.

### 2.9. Immunofluorescence

After dehydrating the livers, spleens, lungs, and kidneys, which were soaked in a 4% paraformaldehyde solution, we embedded them in paraffin and cut them with a thickness of 4 μm into parts. Then, they were incubated with primary anti TRIM3 (1:100) overnight at 4°C and incubated in the dark for 3–5 min at room temperature with fluorescence-labeled secondary antibodies. We stained them with DAPI without sunlight for 1 min and finally sealed them for microscopic examination to collect images after adding quenching agents.

### 2.10. Statistical analysis

The unpaired, two-tailed Student's *t-*test was used for statistical analysis, and the F-test was performed to confirm whether the two populations had homogeneity of variance. Mann-Whitney *U* test was performed for data not normally distributed. For mice survival studies, we generated Kaplan-Meier survival curves and analyzed them with logarithmic rank tests. All statistical analysis was performed by GraphPad Prism 8.0 software. A value of *P* < 0.05 was considered significant.

## 3. Results

### 3.1. TRIM3 expression is decreased in SFTS patients and mouse model

**Figure 1:**
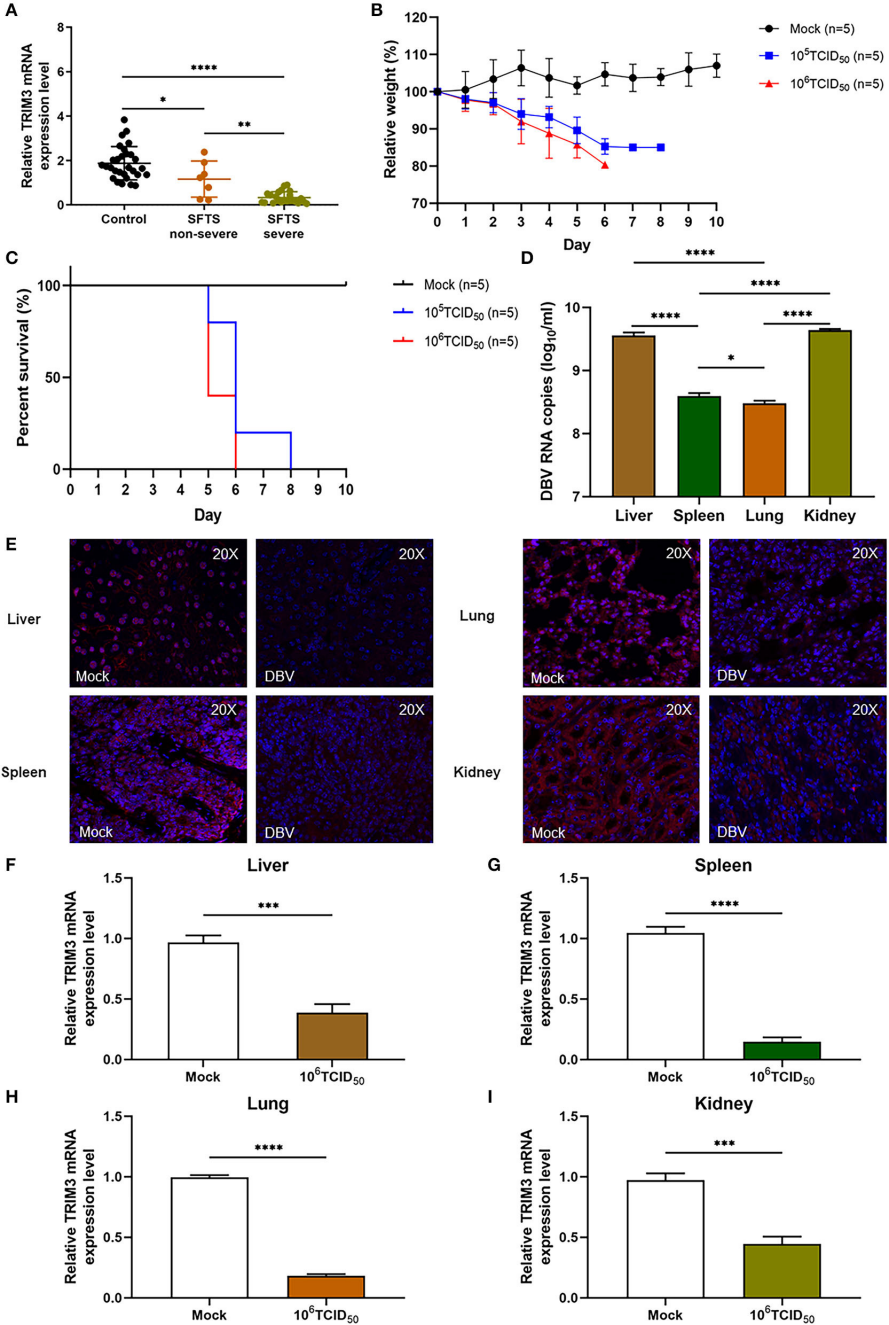
TRIM3 expression is decreased in SFTS patients and mice. **(A)** TRIM3 mRNA expression in PBMC samples of 29 healthy controls, 7 non-severe, and 22 severe SFTS patients. **(B, C)** Body weight and survival of IFNAR^−/−^ mice infected with 10^5^ or 10^6^ TCID_50_ of DBV (*n* = 5). Five mice not infected with DBV served as the control group (mock). Relative weight was calculated, and data are presented as the mean ± SEM. **(D)** DBV RNA levels in different tissues of mice infected with 10^6^ TCID_50_ of DBV. **(E)** Representative images showing TRIM3 expression (red) in different tissues of SFTS mice. **(F–I)** TRIM3 mRNA expression in different regions of SFTS mice (^*^P < 0.05; ^**^P < 0.01; ^***^P < 0.001; ^****^P < 0.0001).

The relative expression level of TRIM3 mRNA in PBMC samples of SFTS patients was significantly lower than that of healthy participants, especially in severe cases (Figure 1A). After subcutaneous injection of DBV, the mice were observed daily, and their body weight was recorded. Infected mice manifested abnormal consciousness, a weakened response to external stimuli, hair loss, and a bowed back. All infected mice showed changes in body weight, and the weight loss in the 10^6^ TCID_50_ group was more significant than that in the 10^5^ TCID_50_ group (Figure 1B). The mice began to die on day 5 after infection, and all the mice in the 10^6^ TCID_50_ group died on day 6 after infection, while all the mice in the 10^5^ TCID_50_ group died on day 7 after infection (Figure 1C). An autopsy showed congestion and tissue swelling in the livers, spleens, lungs, and kidneys, with the 10^6^ TCID_50_ group being more pronounced than the 10^5^ TCID_50_ group. qRT-PCR indicated that DBV had a high level of replication in the livers, spleens, lungs, and kidneys, with the most significant replication in tissues of the livers and kidneys (Figure 1D). Immunofluorescence analysis and qRT-PCR of the 10^6^ TCID_50_ group mice tissues showed a significant decrease in the TRIM3 expression in these organs (Figures 1E–I).

### 3.2. TRIM3 inhibits the pro-inflammatory effects of DBV infection in the cell model

Innate immunity is the first line of defense against pathogen invasion (Chatterjee et al., 2016), and peripheral blood monocytes are the main target cells during DBV infection (Peng et al., 2016). Therefore, human PBMCs, THP-1 cells, and mouse peritoneal macrophages were selected for subsequent experiments. Human PBMCs were extracted and co-cultured with DBV at MOI = 0.5, MOI = 1, and MOI = 2.0 for 2 h. Cell viability was detected by the CCK-8 assay. The PBMC's viability was 98.9, 94.2, and 92.3% when MOI = 0.5, MOI = 1, and MOI = 2.0, respectively. Therefore, MOI = 0.5 was chosen as the working concentration for this experiment.

To verify the expression of TRIM3 in the cell model, qRT-PCR, and Western blot were used to detect the expression level of TRIM3 mRNA and protein after DBV infection with PBMCs. Compared with the mock group, the expression of TRIM3 mRNA, and protein was decreased in the DBV-infected group (data were not shown).

**Figure 2:**
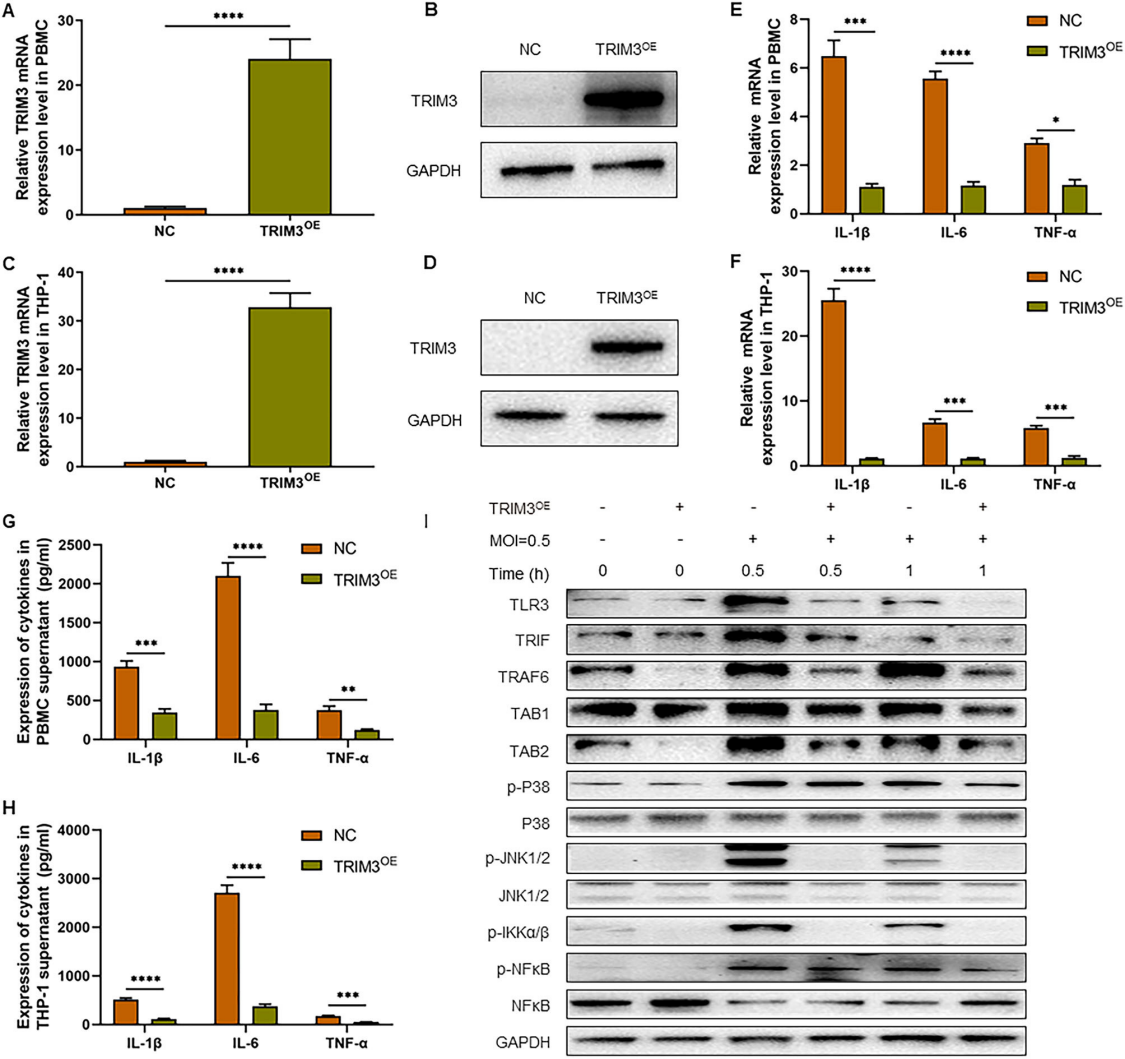
TRIM3 inhibits the pro-inflammatory effects of DBV infection in a cell model. TRIM3 mRNA expression was measured by qRT-PCR in PBMCs **(A)** and THP-1 cells **(C)** transfected with TRIM3^OE^. The TRIM3 protein level was measured by Western blot in PBMCs **(B)** and THP-1 cells **(D)** transfected with the TRIM3^OE^. The mRNA expressions of IL-1β, IL-6, and TNF-α were detected by qRT-PCR in PBMCs **(E)** and THP-1 cells **(F)**. The protein levels of IL-1β, IL-6, and TNF-α in the supernatants of PBMCs **(G)** and THP-1 cells **(H)** were measured by ELISA. **(I)** The total protein in each group of THP-1 cells was extracted to detect TLR3 and downstream pathway activation by Western blot (OE, overexpression; NC, Negative control; ^**^P < 0.01; ^***^P < 0.001; ^****^P < 0.0001).

To confirm the role of TRIM3 in SFTS, we tested whether overexpression of TRIM3 could inhibit DBV-induced inflammation. According to the instructions, we transfected PBMCs and THP-1 cells with lentiviral vectors to overexpress TRIM3. qRT-PCR and Western blot analysis showed that TRIM3 mRNA and protein in PBMCs (Figures 2A, B) and THP-1 cells (Figures 2C, D) increased significantly in the TRIM3^OE^ group. Total mRNA was extracted from cells 24 h after DBV infection. The relative expression levels of IL-1β, IL-6, and TNF-α mRNA in PBMCs and THP-1 cells increased after DBV infection. After overexpression of TRIM3, the relative mRNA expression of the above inflammatory cytokines decreased significantly (Figures 2E, F). Meanwhile, at 24 h after DBV infection, the expressions of IL-1β, IL-6, and TNF-α in the supernatant were significantly increased, while the overexpression of TRIM3 could significantly reduce the secretion of these cytokines (Figures 2G, H).

To further explore the mechanism of increased inflammatory factors, total intracellular proteins were extracted 1 h after DBV infection, and the expression of TLR3 and the activation of the downstream Mitogen-activated protein kinase (MAPK) pathway in THP-1 cells of different groups were detected by Western blot. After DBV infection, TLR3, and MAPK signaling pathways are activated, with p38 and JNK1/2 phosphorylation being more pronounced. After overexpression of TRIM3, TLR3 activation decreased, and the MAPK signaling pathway was significantly inhibited. It is suggested that overexpression of TRIM3 can inhibit the activation of TLR3 and MAPK signaling pathways induced by DBV (Figure 2I).

### 3.3. TRIM3 deficiency exacerbates the pro-inflammatory effects of DBV infection in the cell model

**Figure 3:**
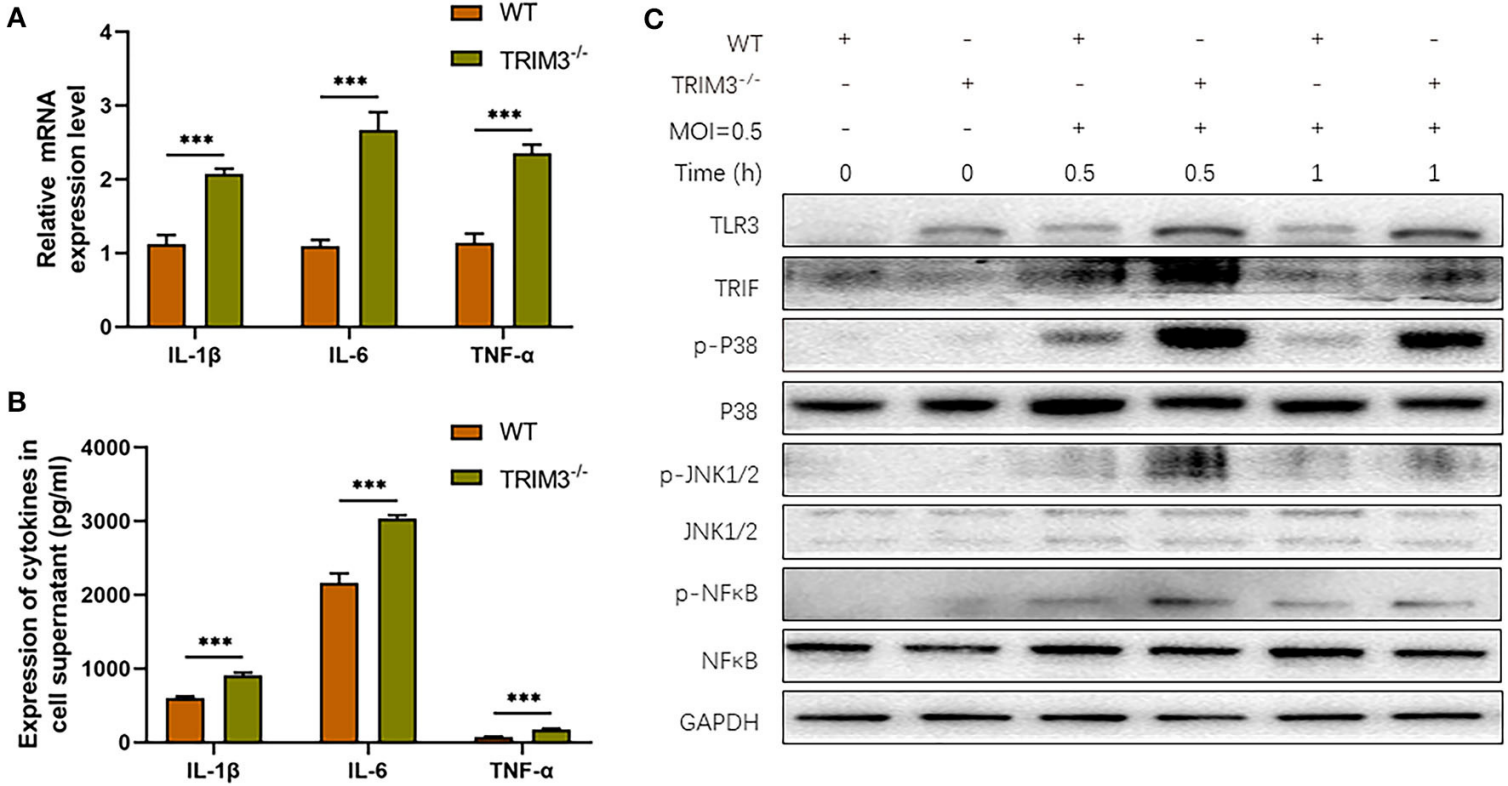
TRIM3 deficiency exacerbates the pro-inflammatory effects of DBV infection in a cell model. **(A)** The mRNA expressions of IL-1β, IL-6, and TNF-α were measured by qRT-PCR in peritoneal macrophages derived from TRIM3^−/−^ and WT mice. **(B)** The protein levels of IL-1β, IL-6, and TNF-α in the supernatants of macrophages were measured by ELISA. **(C)** The total protein in each group of macrophages was extracted to detect TLR3 and downstream pathway activation by Western blot (TRIM3^−/−^, TRIM3 knockout; WT, wild type; ^***^P < 0.001).

We further investigate the effect of TRIM3 deletion on DBV-induced inflammatory signaling pathway activation and cytokines secretion. Peritoneal macrophages from TRIM3^−/−^ and wild-type (WT) mice were extracted. Total mRNA and cell supernatant were collected 24 h after DBV infection. The relative expression levels of IL-1β, IL-6, and TNF-α mRNA increased 24 h after infection, while the mRNA expression of the above cytokines showed a remarkable rise after TRIM3 knockout (Figure 3A). The protein expressions of IL-1β, IL-6, and TNF-α in the supernatant changed synchronously. It was significantly increased 24 h after DBV infection, and knockout of TRIM3 significantly promoted DBV-induced cytokine secretion (Figure 3B). After DBV infection in mouse peritoneal macrophages, TLR3 was activated, and proteins in the MAPK signaling pathway were phosphorylated. TLR3 activation was increased, and protein phosphorylation in the MAPK pathway was significantly promoted in the TRIM3 knockout group. These results suggest that the deletion of TRIM3 can promote the activation of TLR3 and MAPK pathway proteins induced by DBV infection (Figure 3C).

### 3.4. TRIM3 regulates TLR3 involvement in DBV-induced inflammatory activation through K48-linked ubiquitin

**Figure 4:**
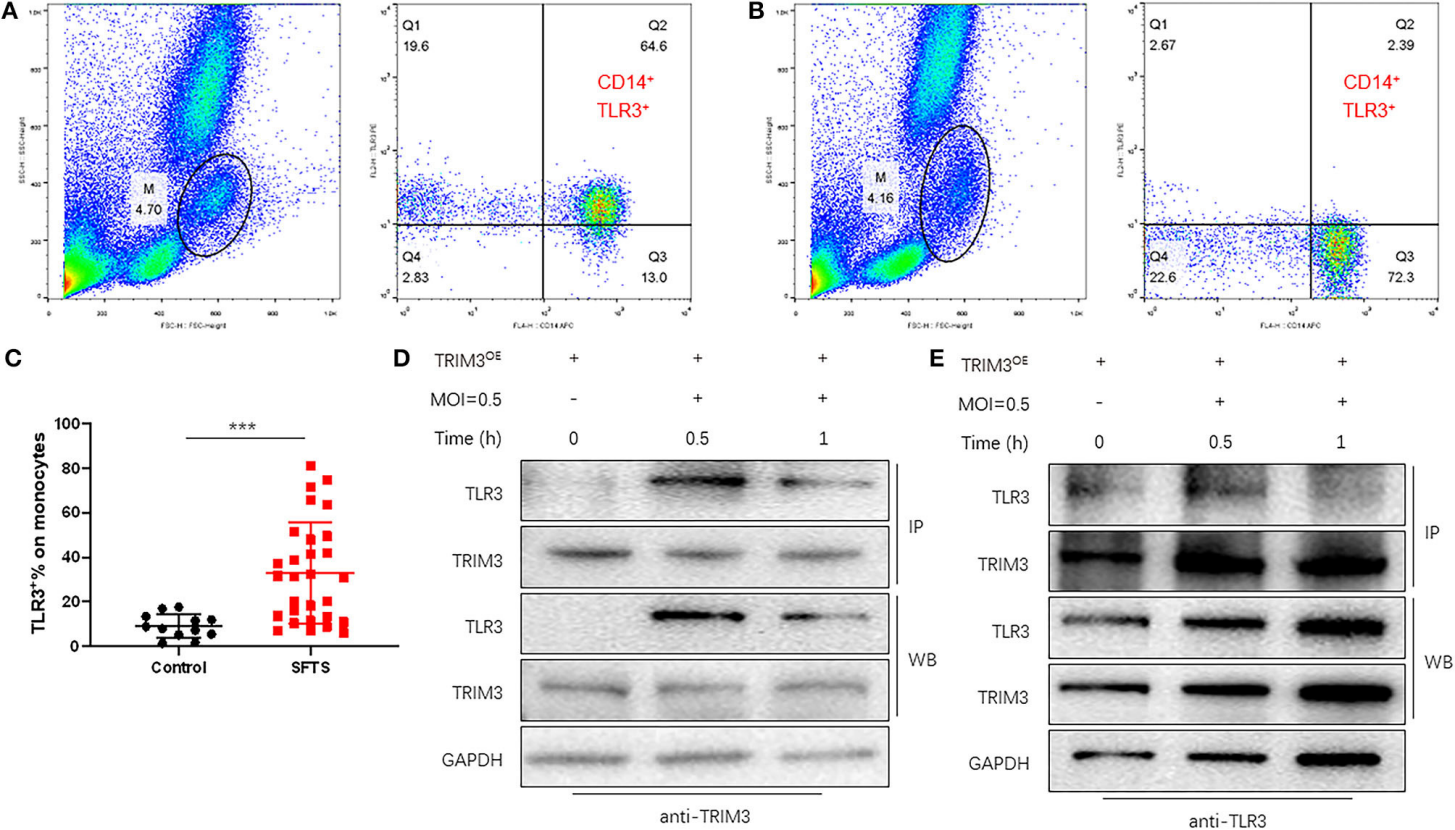
TRIM3 directly binds to TLR3 and activates the TLR3 pathway to produce an anti-inflammatory effect. **(A–C)** The proportion of TLR3-positive cells in monocytes of 29 SFTS patients **(A)** and 12 normal subjects **(B)** was determined by flow cytometry. **(D, E)** Cell lysate from THP-1 cells in each group was detected by IP for TRIM3/TLR3, and eluted proteins were analyzed by Western blot with anti-TRIM3 and anti-TLR3 antibodies (^***^P < 0.001).

TLR3-mediated signaling is important for the innate immune response against RNA viruses (Zang et al., 2020). TLR3 expression was increased in DBV-infected THP-1 cells. To observe the expression of TLR3 in SFTS patients, flow cytometry was used to detect monocytes in 29 patients and 12 normal subjects. It revealed that compared with normal subjects, monocyte activation and TLR3 expression levels were significantly increased in SFTS patients (Figures 4A–C).

**Figure 5:**
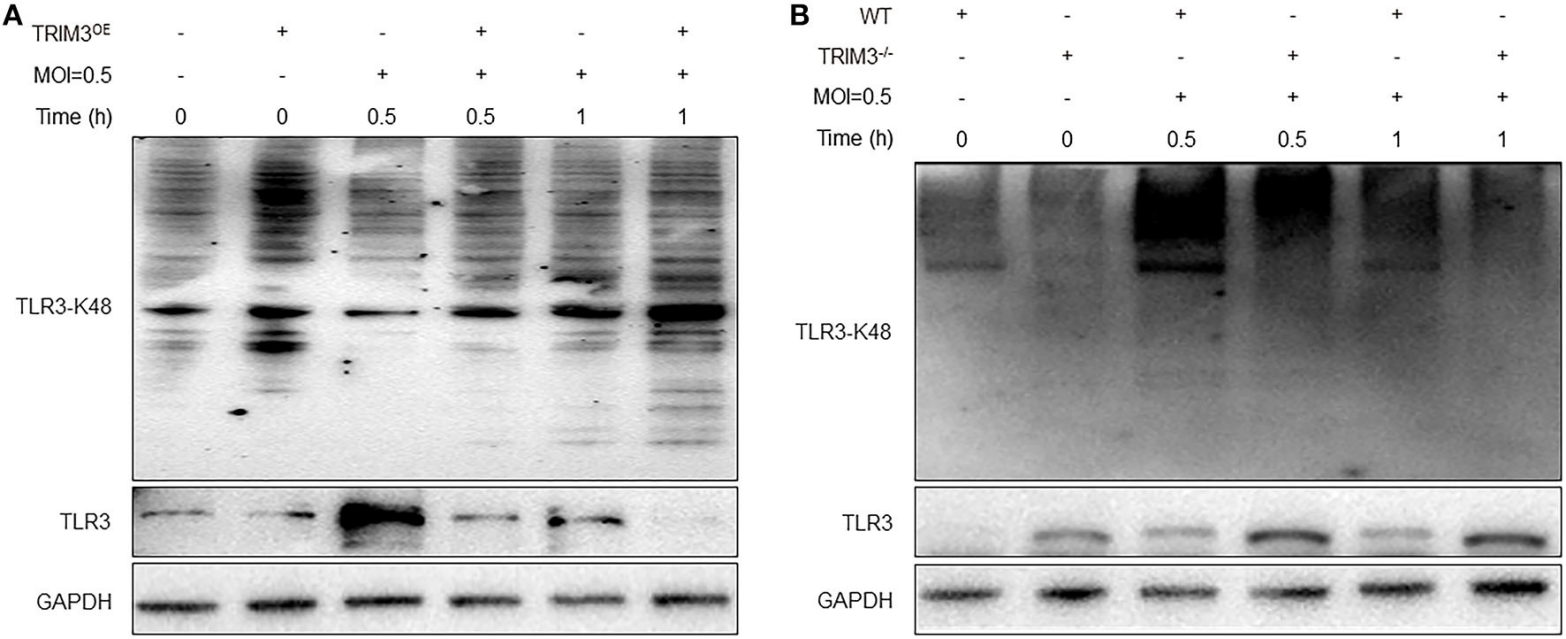
TRIM3 promotes K48-linked ubiquitination and degradation of TLR3. Western blot analysis was performed to evaluate K48-linked ubiquitination and degradation of TLR3 in each group of THP-1 transfected with the TRIM3^OE^ **(A)** and peritoneal macrophages derived from TRIM3^−/−^ and WT mice **(B)**.

TRIM3 is an E3 ubiquitin ligase that can exert its immune regulatory effect by modifying proteins through ubiquitination. TRIM3 can inhibit DBV-induced elevated TLR3 expression, but the specific mechanism remains unclear. Western blot and IP analysis showed that in DBV-infected THP-1 cells with overexpression of TRIM3, DBV infection would lead to corresponding changes in TLR3 along with the change in TRIM3 expression, and there was an interaction between TRIM3 and TLR3 (Figures 4D, E). In DBV-infected TRIM3^OE^ THP-1 cell lines, TLR3 expression levels decreased compared to controls, which was associated with increased K48-linked ubiquitin (Figure 5A). In DBV-infected TRIM3^−/−^ mice's peritoneal macrophages, compared with the WT group, K48-linked ubiquitin was decreased and TLR3 expression was increased (Figure 5B). These results indicate that TRIM3 can bind to TLR3 and degrade TLR3 through K48-linked ubiquitination, which plays a crucial role in inhibiting cytokine storm.

## 4. Discussion

Although there have been many studies related to DBV-induced immune imbalance and cytokine storms, there are still no effective drugs to regulate the immune and inflammatory states of SFTS. This study found that TRIM3 expression is decreased in SFTS patients, especially in severe cases and IFNAR^−/−^ mice model. In DBV-infected THP-1 cells, the overexpression of TRIM3 can inhibit the DBV-induced release of cytokines, the expression of TLR3, and protein phosphorylation in the MAPK pathway, which exerts an anti-inflammatory role. In DBV-infected peritoneal macrophages extracted from TRIM3^−/−^ mice, TRIM3 deficiency exacerbates cytokines release, increases TLR3 expression and protein phosphorylation of the MAPK pathway, and amplifies inflammation caused by DBV. In support of this notion, TLR3 expression was confirmed to be upregulated in the monocytes of SFTS patients. We further investigated whether TRIM3 can interact with TLR3 and promote TLR3 degradation through K48-linked ubiquitination.

The innate immune response is the first line of defense against viral infection. High-load viruses promote an abnormal and uncontrolled innate immune response through the over-activation of TLR signaling pathways, which induce the production of multiple inflammatory cytokines and chemokines and thus triggering fatal SFTS (Yamada et al., 2018). Therefore, elucidating the immune response mechanism after DBV infection is crucial for preventing the occurrence and progression of severe SFTS cases. Inflammation is an external manifestation of the immune system. Our results showed that DBV induced the release of pro-inflammatory cytokines including IL-1β, IL-6, and TNF-α. Li Z. et al. (2022) also indicated IL-1β secretion was elevated in SFTS patients and infected mice, and IL-1β level in blood was reversibly correlated with disease severity and viral load in SFTS patients. IL-1β not only activates innate immune cells, such as monocytes, macrophages, and neutrophils but also drives the development of helper CD4^+^ T cells by regulating Th1 and Th17, which activate adaptive immunity subsequently (Cheung et al., 2018).

Our results revealed that TRIM3 plays an immunomodulatory role in DBV infection by inhibiting the MAPK signaling pathway. The effects of TRIMs on virus replication, recognition, and clearance have become a hot topic in the field of infection immunology in recent years. In human microglia, TRIM21 down-regulates IFN-β production induced by Japanese encephalitis virus (JEV) infection, thereby reducing JEV-mediated pathological effects (Manocha et al., 2014), and TRIM52 can inhibit JEV replication (Fan et al., 2016). TRIM7 can also decrease the replication of the encephalomyocarditis virus by activating the IFN-β signaling pathway (Li M. et al., 2023). Furthermore, many researchers have reported the crucial role of the TRIM protein family in the MAPK pathway. TRIM38 interacts with TRAF6 and TRIF and mediates the K48-linked polyubiquitination of TRAF6 and TRIF to promote their degradation (Hu et al., 2015). TRIM8 can activate the K63-linked polyubiquitination of TAK1 to enhance the activation of NF-κB in *Pseudomonas aeruginosa*-induced keratitis (Guo et al., 2017). Lou et al. (2018) also demonstrated that TRIM22 can regulate autophagy *via* the NF-κB/beclin 1 pathway and prevent the autophagic clearance of *Mycobacterium tuberculosis* in THP-1 cells. TRIM36 can enhance the suppression effect of anti-androgen on prostate cancer by reducing the phosphorylation of proteins in the MAPK pathway (Liang et al., 2018). TRIM3, as a member of the TRIM family, also regulates immune and inflammatory responses. TRIM3 overexpression has an inhibitory effect on the proliferation and cytokine secretion of fibroblast-like synoviocytes in rheumatoid arthritis (Wang et al., 2019) and can protect against LPS-induced acute kidney injury by inhibiting the IRF3 pathway and NLRP3 inflammasome activation (Li W. et al., 2022). TRIM3^−/−^ cells and mice also expressed lower levels of antiviral genes and exhibited lower levels of inflammation response following poly (I:C) but not the stimulation of lipopolysaccharide (Li et al., 2020). However, no research has reported the changes and effects on the immune and inflammatory responses of TRIM3 *in vitro* and *in vivo* after DBV infection so far. In this study, we found that TRIM3 is downregulated in PBMCs of SFTS patients and organ tissues of the SFTS mice model, and its overexpression improves the DBV-induced inflammatory cytokine storm by inhibiting the MAPK pathway, which suggests that TRIM3 may be a potential target for controlling the development and progression of SFTS.

TLR3 is a pattern recognition receptor used to sense viral nucleic acid, relies on TRIF protein to activate downstream TAK1, activates the NF-κB and MAPKs cascade pathways, regulates the expression of target genes, and leads to the release of a large number of cytokines, which is critical for the activation of the host immune response to virus infection (Yamashita et al., 2012; Verma and Bharti, 2017). TLR3 is expressed in a variety of cell types, such as dendritic cells, macrophages, and fibroblasts (Yang et al., 2016). In the DBV-infected THP-1 cells, we found that TLR3 expression is elevated, and TRIM3 overexpression can downregulate TLR3 expression while TRIM3 deletion upregulates. Nonetheless, how TRIM3 specifically and efficiently interacts with TLR3 remains poorly understood. Li et al. (2020) also suggested that K63-linked polyubiquitination of TLR3 mediated by TRIM3 is a positive regulatory mechanism for TLR3-mediated innate immune and inflammatory responses. Therefore, the CO-IP experiment further verified the interaction between TRIM3 and TLR3. Subsequently, we confirmed that TRIM3 could degrade TLR3 through K48-linked ubiquitination, which inhibits inflammation induced by DBV infection.

The study has some limitations. First, the exact target of the interaction between TRIM3 and TLR3 is still unclear and needs to be further explored. Second, it is still difficult to construct a lethal model of SFTS mice with overexpression or knockout of the *TRIM3* gene, and it is necessary to further verify the regulatory effects of TRIM3 on inflammatory signaling pathway conduction and cytokine storm *in vivo*. Third, we explored the effects of TRIM3 on the MAPK pathway and downstream pro-inflammatory cytokines, but the influence and mechanism of TRIM3 on anti-inflammatory cytokines such as IL-10 still need to be further studied. Although the TRIM3-TLR3 pathway may mediate the inflammatory regulation effect of TRIM3 in immune cells, it does not rule out the possibility that TRIM3 protects cells from immune damage through other mechanisms.

## 5. Conclusion

This study revealed that TRIM3 attenuates the cytokine storm caused by DBV infection by promoting TLR3 degradation by inhibiting the MAPK signaling pathway, which provides an innovative therapeutic target for improving the prognosis of SFTS.

## Data availability statement

The raw data supporting the conclusions of this article will be made available by the authors, without undue reservation.

## Ethics statement

The studies involving human participants were reviewed and approved by the Ethics Committee of the Institutional Review Board at The First Affiliated Hospital of Nanjing Medical University. The patients/participants provided their written informed consent to participate in this study. The animal study was reviewed and approved by the Huadong Medical Institute of Biotechniques Ethics Committee.

## Author contributions

KJ, YD, JZ, and JL designed the experiments, writing, review, and editing. KJ, YD, KO, HH, ZJ, ZY, TZ, HL, ChunhW, ChunyW, and XS performed the experiments. KJ, YD, KO, DL, XL, NH, and CZ analyzed and interpreted the data from experiments. JZ and JL contributed to funding acquisition and supervision. All authors approved the publication of the manuscript.
