# Supplementary material for: TRIM3 attenuates cytokine storm caused by Dabie bandavirus via promoting Toll-like receptor 3 degradation
Source: Front Microbiol. 2023 Jul 14;14:1209870. doi: 10.3389/fmicb.2023.1209870 (PMC10375709; doi:10.3389/fmicb.2023.1209870)
Supplement: Supplementary file 1 [file Table_1.PDF]

Supplementary Material

TRIM3 attenuates cytokine storm caused by Dabie bandavirus via promoting Toll-like receptor 3 degradation

Ke Jin[1†], Yan Dai[1†], Ke Ouyang[1], Huaying Huang[1,2], Zhengyi Jiang[1], Zhan Yang[3], Tingting Zhou[3], Hong Lin[4], Chunhui Wang[3], Chunyan Wang[3], Xuewei Sun[5], Dafeng Lu[6], Xiaoguang Liu[7], Nannan Hu[1], Chuanlong Zhu[1], Jin Zhu[3*], Jun Li[1*]

[†] These authors contributed equally to this work and share first authorship.

*** Correspondence:**

Jin Zhu, Huadong Medical Institute of Biotechniques, Nanjing 210018, China. Email: zhujin1968@njmu.edu.cn

Jun Li, Department of Infectious Disease, the First Affiliated Hospital of Nanjing Medical University, Nanjing 210029, China. Email: dr-lijun@vip.sina.com

1 Supplementary Tables

1.1 Supplementary Tables

Supplementary Table 1. Clinical characteristics of patients with severe fever with thrombocytopenia syndrome.

**Case No.**	**Age/sex**	**Days of onset**	**Severity**	**Stage**	**Blood DBV load (lg copies/ml)**	**Outcome**
1	46/M	6	Non-severe	Fever	5.50	Survived
2	69/M	5	Severe	Fever	5.84	Survived
3	55/M	14	Non-severe	MOF	3.03	Survived
4	67/F	10	Severe	MOF	5.97	Survived
5	79/F	9	Severe	MOF	4.87	Survived
6	56/M	6	Non-severe	MOF	5.92	Survived
7	67/F	5	Severe	MOF	6.43	Survived
8	76/F	8	Severe	MOF	7.72	Survived
9	67/F	7	Non-severe	MOF	3.66	Survived
10	71/F	14	Severe	MOF	8.84	Survived
11	69/F	9	Non-severe	MOF	5.22	Survived
12	48/M	9	Severe	MOF	5.82	Survived
13	71/M	10	Severe	MOF	6.88	Survived
14	68/M	6	Non-severe	MOF	6.57	Survived
15	78/M	17	Severe	MOF	7.30	Survived
16	70/M	17	Non-severe	MOF	4.43	Survived
17	66/F	9	Severe	MOF	6.19	Survived
18	49/M	9	Severe	MOF	5.34	Survived
19	59/M	13	Severe	MOF	7.58	Survived
20	52/F	9	Severe	MOF	7.03	Survived
21	65/F	18	Severe	MOF	5.79	Survived
22	77/M	10	Severe	MOF	6.67	Died
23	78/M	10	Severe	MOF	6.96	Died
24	65/M	14	Severe	MOF	5.92	Survived
25	73/M	8	Severe	MOF	6.56	Died
26	72/M	10	Severe	MOF	6.90	Survived
27	79/M	4	Severe	Fever	6.54	Died
28	74/M	5	Severe	MOF	8.90	Died
29	74/M	6	Severe	MOF	9.12	Died

MOF, multiple organ failure.

Supplementary Table 2. Primers in qRT-PCR analysis.

Gene	Primer Sequence (5'-3')
TRIM3	**F: 5'** CTGTGAGACGGCCATGTGT **3'**
	**R: 5'** AAATGGTCTCCAGGTCGCTG **3'**
IL-1β	**F: 5'** ATGATGGCTTATTACAGTGGCAA **3'**
	**R: 5'** GTCGGAGATTCGTAGCTGGA **3'**
IL-6	**F: 5'** ACCCCCAGGAGAAGATTCCA **3'**
	**R: 5'** TCACCAGGCAAGTCTCCTCA **3'**
TNF-α	**F: 5'** GCTGCACTTTGGAGTGATCG **3'**
	**R: 5'** TATCTCTCAGCTCCACGCCA **3'**
GAPDH	**F: 5'** ACAACTTTGGTATCGTGGAAGG **3'**
	**R: 5'** GCCATCACGCCACAGTTTC **3'**

F, forward; R, reverse.
